# Mechanical properties of sintered meso-porous silicon: a numerical model

**DOI:** 10.1186/1556-276X-7-597

**Published:** 2012-10-29

**Authors:** Roberto Martini, Valerie Depauw, Mario Gonzalez, Kris Vanstreels, Kris Van Nieuwenhuysen, Ivan Gordon, Jef Poortmans

**Affiliations:** 1, Department of Electrical Engineering (ESAT), KU Leuven, Kasteelpark 10, Leuven-Heverlee 3001, Belgium; 2, Interuniversitair Micro-Electronica Centrum (IMEC), Kapeldreef 75, Leuven-Heverlee 3001, Belgium

**Keywords:** Porous silicon, Homogenization, Irregular microstructure, Finite element method

## Abstract

Because of its optical and electrical properties, large surfaces, and compatibility with standard silicon processes, porous silicon is a very interesting material in photovoltaic and microelectromechanical systems technology. In some applications, porous silicon is annealed at high temperature and, consequently, the cylindrical pores that are generated by anodization or stain etching reorganize into randomly distributed closed sphere-like pores. Although the design of devices which involve this material needs an accurate evaluation of its mechanical properties, only few researchers have studied the mechanical properties of porous silicon, and no data are nowadays available on the mechanical properties of sintered porous silicon. In this work we propose a finite element model to estimate the mechanical properties of sintered meso-porous silicon. The model has been employed to study the dependence of the Young’s modulus and the shear modulus (upper and lower bounds) on the porosity for porosities between 0% to 40%. Interpolation functions for the Young’s modulus and shear modulus have been obtained, and the results show good agreement with the data reported for other porous media. A Monte Carlo simulation has also been employed to study the effect of the actual microstructure on the mechanical properties.

## Background

Porous silicon (PSi) has been extensively employed in microelectromechanical systems (MEMS) technology and it has been proposed for some applications in photovoltaics (PV) technology. In MEMS technology, processes to manufacture suspended structures often employ open-porosity PSi as a sacrificial layer while in PV technology, stacked layers of sintered PSi with different porosity have been proposed both as buried Bragg reflectors and for layer transfer techniques for the fabrication of thin silicon solar cells [1]. In these applications, an accurate evaluation of PSi mechanical properties is paramount for the device fabrication and performance.

Since its discovery, PSi has been investigated mostly for its optical and electrical properties. Only few researchers investigated the mechanical properties of this material. Characterizations of PSi mechanical properties were performed employing very different techniques, e.g., nanoindentation [2], Brillouin scattering [3], phase velocity scanning [4], and microechography [5], but each of these works dealt with open-porosity PSi.

**Figure 1:**
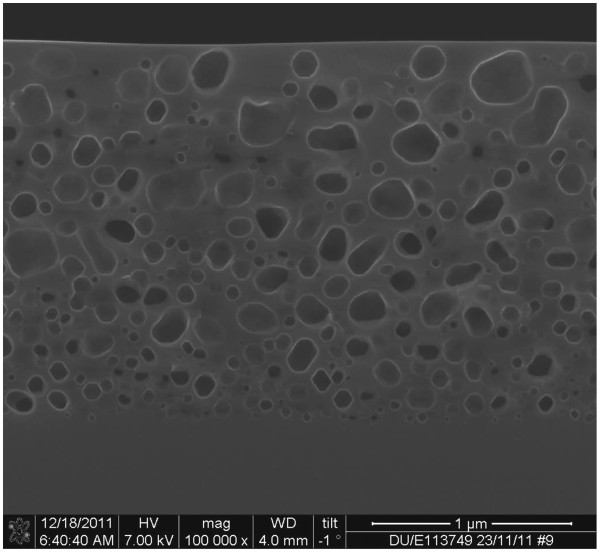
**SEM picture of a sintered PSi cross section.** SEM picture of sintered meso-PSi after 20 min annealing in H_2_ atmosphere at 1,100°C and 1 atm.

For some applications such as the layer transfer technique and the buried Bragg reflectors, as-anodized PSi undergoes a thermal treatment which transforms the structure of the silicon layer. When annealed at temperatures higher than 1,000°C, PSi experiences a structural reorganization driven by surface energy minimization which transforms the columnar voids formed by the anodization into closed sphere-like pores. The pores interact and give rise to more complex structures (see Figure 1). To the knowledge of the authors, no studies have been performed about the mechanical properties of the sintered meso-porous silicon. Magoariec and Danescu [6] proposed a numerical model for evaluating the mechanical properties of sintered nano-PSi which they compared with experimental data from literature, but they did not take into account the effect of the random distribution of voids. Li et al. [7] showed that the mechanical properties of porous media depend on the connectivity of the pores and, thus, the actual distribution of voids is expected to play a role in the mechanical properties of porous silicon.

In this work we present a finite element model for the evaluation of the overall mechanical properties of sintered PSi. This numerical model includes a random distribution of voids inside a representative volume element (RVE), and it has been exploited to evaluate the upper and lower bounds of the Young’s modulus and the shear modulus of PSi as a function of porosity. A Monte Carlo simulation is also presented in this work to evaluate the fluctuation of the upper bounds due to the statistical variation of the microstructure.

## Methods

**Figure 2:**
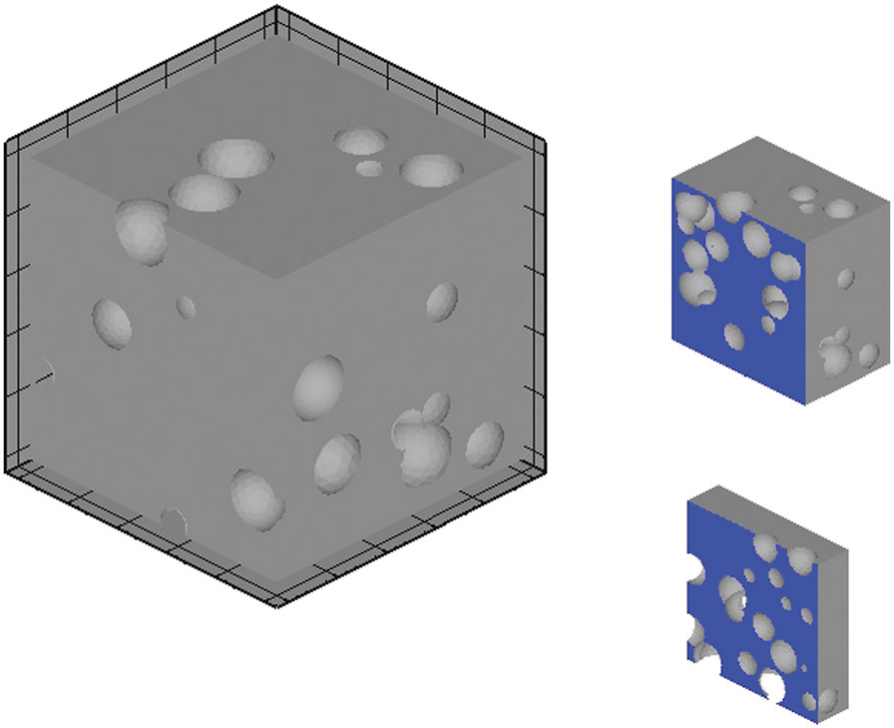
**Example of RVE.** Example of RVE employed for the study of the overall mechanical properties of porous silicon and two cross-sections.

An example of the numerical model employed for the analysis of the mechanical properties of PSi is depicted in Figure 2. The RVE is generated by subtracting a random distribution of spherical voids to a silicon cube. Without loss of generality, we assume that the edges of the cube are oriented along the <100> directions of the silicon crystal lattice. Referring to this orientation, the Young’s modulus, the Poisson ratio and the shear modulus of silicon employed in the analysis are the ones reported by Masolin et al. [8], i.e. *E*_Si_=130 GPa, *ν*_Si_=0.278 and *G*_Si_=79.6 GPa. The position of the center of the voids and their radii follow a rectangular and a Gaussian distribution, respectively. Since the single void can be centered in any location in the silicon cube, there is a non-null probability that two or more voids overlap. The RVEs are then discretized by means of simplicial meshes.

To define the overall mechanical properties of PSi, the standard homogenization theory has been employed. In this framework, three different kinds of boundary conditions are usually applied: uniform displacements, uniform tractions, and periodic boundary conditions. If the considered RVE is sufficiently large, these boundary conditions lead to the same overall mechanical response, but such RVE is usually excessively large to be solved by numerical simulations. For smaller RVEs, uniform tractions and uniform displacements boundary conditions are known to evaluate respectively the upper and lower bounds of the stiffness matrix of the RVE, while periodic boundary conditions give a more reasonable estimation of the homogenized stiffness matrix. In this work we want to focus only on the values that bound the mechanical properties and, thus, periodic boundary conditions will not be taken into account.

In the case of uniform displacement boundary conditions, displacements *u*_*i*_ have been imposed at the boundaries (*∂V*) of the RVE (*V *) by the relation:

$$u_{i}=E_{\mathrm{ij}}x_{j}\;\text{for}\;x\in \mathrm{\partial V}\;,$$

where *E*_*ij*_ are the components of the second order macroscopic strain tensor while, in case of uniform tractions boundary conditions, uniform surface loads have been applied at the boundary of the RVE. In the latter case, the components of the macroscopic strain tensor are computed from the average of the displacement field at the boundaries. Since the aspect ratio of the structures inside the RVE is limited and since, at room temperature, silicon behaves as a linear elastic material, nonlinear effects can be neglected until the stress field locally reaches the strength of silicon.

The displacements and the loads imposed at the boundary induce a stress field inside the matrix that can be described by the second order microscopic stress tensor *σ*_*lm*_(***x***). The second order macroscopic stress tensor *Σ*_*lm*_is evaluated as the average of the microscopic stress field in the RVE:

$$\Sigma_{\mathrm{lm}}=\frac{1}{\left|V\right|}\underset{V}{\int }\sigma_{\mathrm{lm}}(x)\mathrm{dV}\;,$$

where |*V*| denotes the volume of the RVE.

Once the components of the macroscopic stress tensor are computed, the components of the fourth order stiffness tensor (*S*_*ijlm*_) can be extracted by the appropriate ratio between *E*_*ij*_and *Σ*_*lm*_, following the relation:

$$\Sigma_{\mathrm{lm}}=S_{\mathrm{lmij}}E_{\mathrm{ij}}\;.$$

Since bulk silicon shows an orthotropic behavior with equivalent directions along < 100 >, a similar behavior is assumed for the RVE. By making this assumption, the equivalent Young’s modulus and shear modulus in the < 100 > directions can be computed. It has to be noted that the properties obtained by assuming an orthotropic material automatically degenerate in isotropic condition whenever the RVE is not anisotropic.

To study the effect of the actual microstructure on the overall mechanical properties, a Monte Carlo simulation has been performed by generating different realizations with the same statistical distribution of pores positions and radii.

## Results and discussion

**Figure 3:**
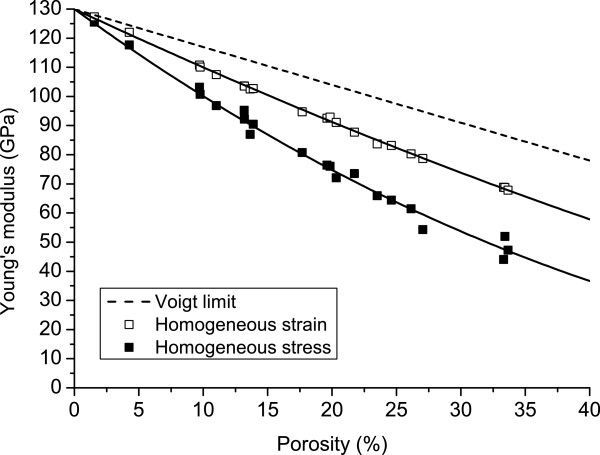
**PSi Young’s modulus as function of porosity.** Numerical results for uniform displacements (empty squares) and uniform traction (full squares) conditions and interpolations (solid line) representing the upper and lower bounds of the homogenized Young’s modulus as function of porosity. Curves are always underneath the Voigt theoretical upper bound (dashed line).

**Figure 4:**
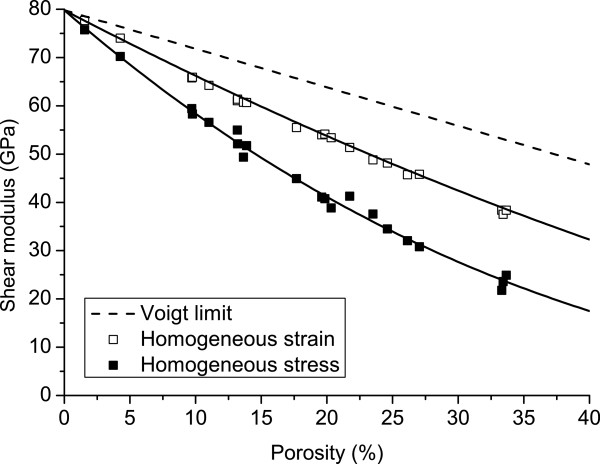
**PSi shear modulus as function of porosity.** Numerical results for uniform displacements (empty squares) and uniform traction (full squares) conditions and interpolations (solid line) representing the upper and lower bounds of the homogenized shear modulus as function of porosity. Curves are always underneath the Voigt theoretical upper bound (dashed line).

Using the method presented in the previous section, the upper and the lower bounds of both the Young’s modulus and the shear modulus of 500×500×500 nm^3^ PSi cubes have been evaluated between 0% and 40% porosities. Evaluations of the Young’s modulus and the shear modulus obtained by this procedure and their interpolations are depicted respectively in Figures 3 and 4. The interpolation functions have the form *A*(*Ψ*)=*A*_Si_×(1−*Ψ*)^*k*^ where *A*(*Ψ*) is the mechanical parameter as function of porosity *Ψ*, *A*_Si_ is the mechanical parameter of the matrix, i.e. silicon, and *k* is the only fitting parameter that has to be tuned in the interpolation. This family of functions is commonly employed for the interpolation of mechanical properties of porous solids [9] and, in this work, they have been employed both for the upper bounds (UBs) and the lower bounds (LBs). The interpolating functions and the relative *R*^2^ values obtained for the Young’s modulus are as follows:

$$\left\{\begin{array}{c} E_{\mathrm{UB}}(\Psi )=E_{\mathrm{Si}}\times {\left(1-\Psi \right)}^{1.58504}\;R^{2}=0.9988\\ E_{\mathrm{LB}}(\Psi )=E_{\mathrm{Si}}\times {\left(1-\Psi \right)}^{2.47781}\;R^{2}=0.9894 \end{array}\right)\;,$$

while for the shear modulus,

$$\left\{\begin{array}{c} G_{\mathrm{UB}}(\Psi )=G_{\mathrm{Si}}\times {\left(1-\Psi \right)}^{1.77023}\;R^{2}=0.9988\\ G_{\mathrm{LB}}(\Psi )=G_{\mathrm{Si}}\times {\left(1-\Psi \right)}^{2.96941}\;R^{2}=0.9922 \end{array}\right)\;.$$

As the *R*^2^values suggest, these functions fit well the mechanical properties obtained by the simulations.

The computed values are compared with the Voigt bound that is known to define the theoretical upper bound for the elastic moduli, while the Reuss lower bound is neglected since it is trivially null. As expected from the homogenization theory, the values gathered from simulations are between the theoretical bounds.

**Figure 5:**
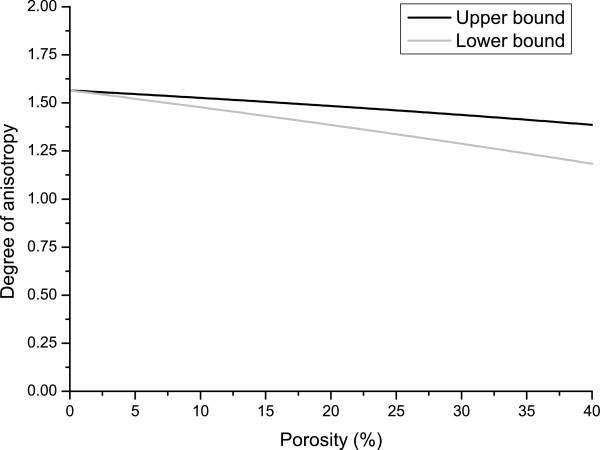
**PSi degree of anisotropy as function of porosity.** Degree of anisotropy computed as $G(\Psi )\frac{2(1+\nu (\Psi ))}{E(\Psi )}$ for both the upper and lower bounds as function of porosity.

In Figure 5, the degree of anisotropy computed as $G(\Psi )\frac{2(1+\nu (\Psi ))}{E(\Psi )}$, i.e., the ratio between the computed shear modulus and the one evaluated by accounting silicon as an isotropic material is reported for both the upper bound and the lower bound. Even though the degree of anisotropy tends to the unity and, therefore, to the isotropic behavior, sintered PSi still behaves as an orthotropic material also for relatively high porosities.

**Figure 6:**
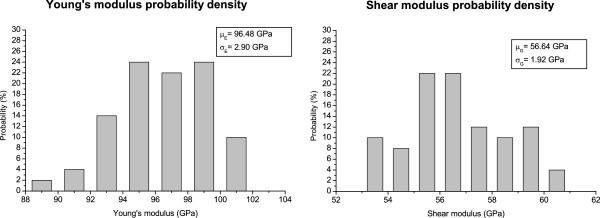
**Statistical distribution of PSi Young’s modulus and shear modulus.** Statistical distribution of PSi Young’s modulus (left) and shear modulus (right) obtained by Monte Carlo simulations. The mean value and the standard deviation are reported in the legend.

Since each realization of the Monte Carlo simulation is generated by keeping constant the statistical distribution of the pores radii and positions, the porosity cannot be also fixed. An *a-posteriori* analysis reveals that the porosity ranges between 15% and 20% with a mean value of 17.66%. Figure 6 shows the statistics of the upper bound of both the Young’s modulus and the shear modulus. Within the legend, the mean values and the variances are reported. To discern the effect of the actual pores distribution from the random change of porosity, the correlation coefficients (*r*_*EΨ*_ and *r*_*GΨ*_) between the logarithm of the two moduli and the logarithm of (1−*Ψ*) have been computed. Both the correlation coefficients (*r*_*EΨ*_=0.9485 and *r*_*GΨ*_=0.9773) report a strong correlation between the porosity and the moduli. This suggests that, once the statistical pores distribution is fixed, the actual microstructure has secondary effect compared to small variations of porosity.

## Conclusions

In this work we presented a finite element model to evaluate the overall mechanical properties of sintered meso-PSi. This model has been employed to characterize the upper and lower bound of the Young’s modulus and shear modulus for meso-PSi with porosity between 0% and 40%. The values defined by the simulations can be fitted by interpolation functions that are commonly employed for porous media. This analysis reduces the theoretical bounds on the mechanical properties defined by the Voigt and Reuss limits and provides an indication on the possible Young’s modulus and shear modulus of sintered PSi as function of porosity. The values of Young’s modulus and shear modulus obtained by simulations are well fitted by interpolation functions that have been already employed for other porous media. This suggests that the model could represent well the actual properties of sintered PSi.

Monte Carlo simulations have also been employed to analyze the effect of the actual microstructure on the upper bound for porosities between 15% and 20%. The results show that the large spread on the values of the Young’s modulus and shear modulus is mainly due to the variation of the porosity instead of the variation of the actual microstructure itself.

The obtained values can be employed for the optimization of structures which involve sintered PSi, and the model can be exploited to study sintered PSi with different pores distributions.

## Competing interests

The authors declare that they have no competing interests.

## Authors’ contributions

RM developed the finite element code, ran the simulations and wrote the manuscript. VD, MG, KV, KVN, IG and JP helped in designing the work and proofreading the manuscript. All authors read and approved the final manuscript.

## Authors’ information

RM is PhD student at KU Leuven. VD is a research engineer in thin-film silicon solar cells at IMEC. MG is a research engineer in packaging reliability at IMEC. KV is a research engineer in packaging reliability at IMEC. KVN is a research engineer in thin-film silicon solar cells at IMEC. IG is a senior researcher at IMEC. JP is a professor at ESAT Department of KU Leuven and the photovoltaics program director at IMEC.
